# The esperanza window trap reduces the human biting rate of *Simulium ochraceum* s.l. in formerly onchocerciasis endemic foci in Southern Mexico

**DOI:** 10.1371/journal.pntd.0005686

**Published:** 2017-07-07

**Authors:** Mario A. Rodríguez-Pérez, Javier A. Garza-Hernández, Mario C. Salinas-Carmona, Ildefonso Fernández-Salas, Filiberto Reyes-Villanueva, Olga Real-Najarro, Eddie W. Cupp, Thomas R. Unnasch

**Affiliations:** 1 Centro de Biotecnología Genómica, Instituto Politécnico Nacional, Reynosa, Tamaulipas, México; 2 Laboratorio de Biologia Molecular, Universidad Autonoma Agraria Antonio Narro, Unidad Laguna, Torreon, Coahuila. C. P., México; 3 Universidad Autónoma de Nuevo León, Facultad de Medicina, Monterrey, Nuevo León, México; 4 Centro Regional de Investigación en Salud Pública, Instituto Nacional de Salud Pública, Tapachula, Chiapas, México; 5 Consejería de Educación, Madrid, España. Calle Maestro 19, Leganés (Madrid) Madrid, España; 6 Department of Entomology and Plant Pathology, Auburn University, Auburn, Alabama, United States of America; 7 Global Health Infectious Disease Research Program, Department of Global Health, University of South Florida, Tampa, Florida, United States of America; Yale School of Public Health, UNITED STATES

## Abstract

**Background:**

The Esperanza Window Trap (EWT) baited with CO_2_ and human sweat compounds is attractive to *Simulium ochraceum* s.l., the primary vector of *Onchocerca volvulus* in the historically largest endemic foci in México and Guatemala.

**Methodology/Principal findings:**

The ability of the EWT to locally reduce numbers of questing *S*. *ochraceum* s.l. was evaluated in two formerly onchocerciasis endemic communities in Southern México. At each community, two EWTs were placed in or near a school or household and flies were collected sequentially for a total of 10 days. Black fly collections were then carried out for an additional 10 days in the absence of the EWTs. Flies were also collected outside the dwellings to control for variations in the local fly populations. When the EWTs were present, there was a significant reduction in the human biting rate at both the household and school locations at collection sites, with a greater effect observed in the schools.

**Conclusions/Significance:**

These results indicate that the EWTs not only have potential as a black fly monitoring tool but may be used for reducing personal exposure to fly bites in Mesoamerica.

## Author summary

The Esperanza window trap (EWT), when used in large numbers (3-4/household and >90% coverage) and baited with human sweat compounds and CO_2_, can be used to collect epidemiologically significant numbers of *Simulium ochraceum* s.l., the primary vector of *Onchocerca volvulus* in the historically largest endemic foci in México and Guatemala. In the present study, we evaluated the ability of the EWT to reduce the personal biting rate by questing *S*. *ochraceum* s.l. in two formerly onchocerciasis endemic communities in Southern México. At each community, two EWTs were placed in or near a school or household and flies were collected sequentially for a total of 10 days. Black fly collections were then carried out for an additional 10 days in the absence of the EWTs. When the EWTs were present, there was a significant reduction in the human biting rate at each of the four collection sites, varying from 14% to 51%. This study demonstrates that the EWTs have potential both as a black fly monitoring and personal exposure reduction tool in Mesoamerica.

## Introduction

The species belonging to the genus *Simulium*, commonly known as black flies, (Diptera: Simuliidae), serve as vectors of several pathogens including nematode parasites, protozoans and viruses [1]. Until recently, *Simulium ochraceum* s.l. was the principal vector of the filarial parasite *Onchocerca volvulus* (the causative agent of river blindness or onchocerciasis) in Latin America and accounted for approximately 70% of the transmission in this region [2]. *Simulium damnosum* s.l. is the primary vector species group of *O*. *volvulus* in sub-Saharan Africa [3]. If untreated, onchocerciasis remains a serious public health concern [4] and poses an enormous source of productivity loss in many African counties [5]. The Onchocerciasis Elimination Program for the Americas (OEPA), has eliminated onchocerciasis throughout in four of the six endemic countries of Latin America, employing a strategy primarily based upon twice per year community wide mass treatments with Mectizan (ivermectin) [6]. African national and international programs are attempting to replicate this success in Africa, again relying primarily on community-wide treatment of the endemic populations with Mectizan [7]. However, if an efficacious and cost-effective method to reduce human vector contact could be added, then the process of parasite elimination could be expedited.

Recently we reported the development of a trap (known as the Esperanza Window Trap, or EWT) for the purpose of replacing human landing collections (HLCs) for monitoring and surveillance of *O*. *volvulus* transmission [8–11]. During field evaluations of the EWT, we observed that while black flies were attracted to workers that were setting up and maintaining the traps, the flies frequently diverted from those individuals and chose to land on the trap. This suggested that the EWTs might be useful in reducing human landing rates (which approximate the biting rate; hence, this term will be used from now on) when deployed in areas frequented by people during their normal daily activities. To test this hypothesis, we evaluated the ability of the EWT to reduce human biting rates in elementary schools and households in Las Golondrinas, and Jose Maria Morelos, two communities located in Southern Chiapas, México.

## Materials and methods

### Ethics statement

The HLC protocols were reviewed and approved by the Bioethics Committees of the Center for Research and Development in Health Sciences of the Autonomous University of Nuevo León (Monterrey, Nuevo León, México). Written informed consent was obtained from the fly collectors, the teachers of the elementary schools and from the owners of the households.

### Study area

**Fig 1:**
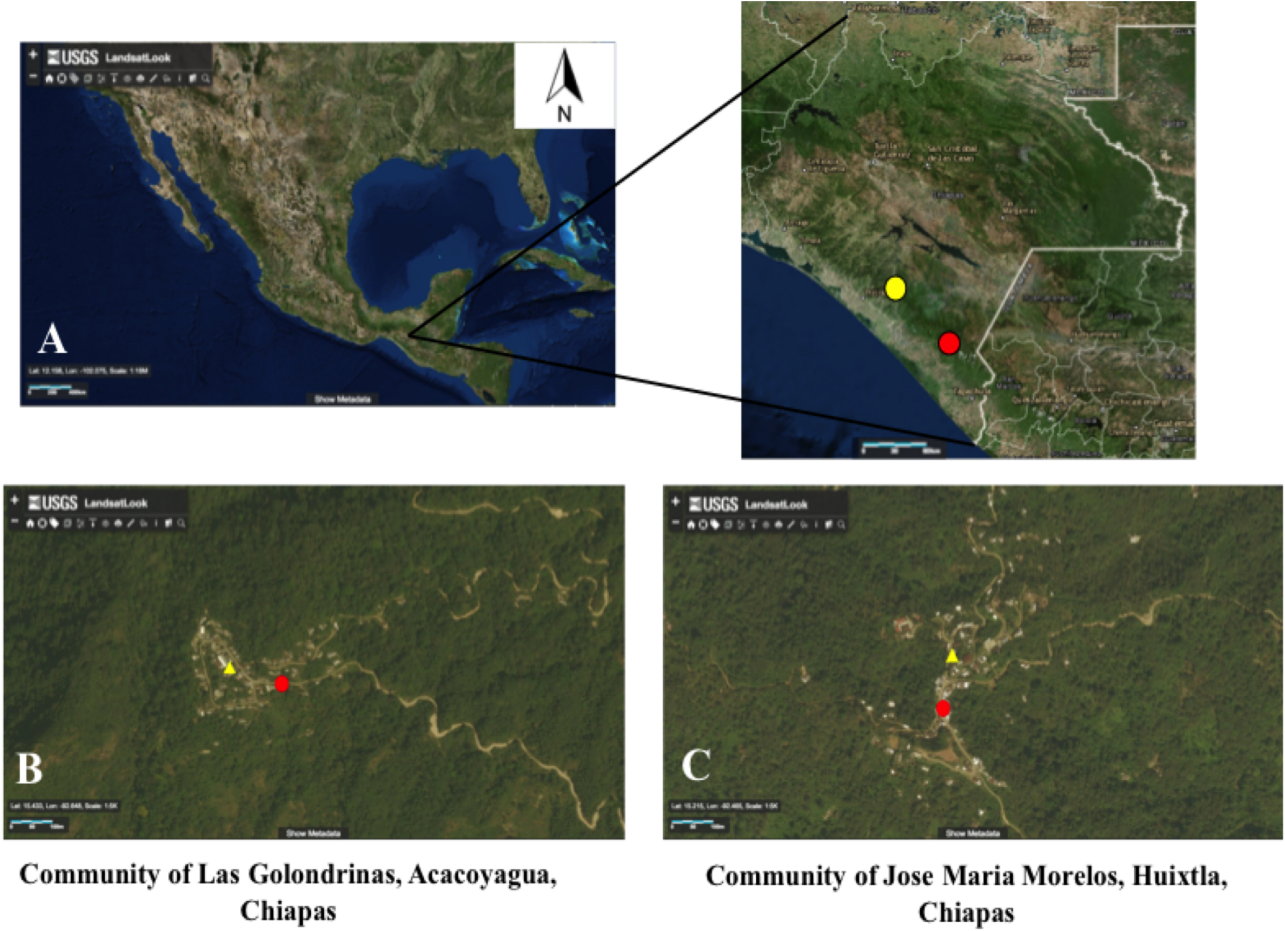
Communities of Las Golondrinas and Jose Maria Morelos. (A) Location of the communities of Las Golondrinas, and Jose Maria Morelos. (B) Location of household and school study sites in Las Golondrinas. (C) Location of household and school study sites in Jose Maria Morelos. On Fig 1B and C, red circles indicate the location of the elementary school and yellow triangle the location of the household. Source: https://landsatlook.usgs.gov/viewer.html.

The study was conducted in an elementary school and a typical household of the communities of Las Golondrinas (Latitude 15°25´ 56.24580˝ N; Longitude 92°39´ 15.21698˝ W, elevation 862 m), and Jose Maria Morelos (Latitude 15°13´ 01.56 ˝ N; Longitude 92°28´ 20˝ W, elevation 1360 m) (Fig 1) and were carried out during the dry season from March throughout May 2016. These communities are located in Southern Chiapas, México. Las Golondrinas is located within the El Triunfo Natural Reserve while Jose Maria Morelos is located 90 km away from the municipality of Huixtla. The population of each village at the time that the study was carried out was 360 and 340 inhabitants respectively. Transmission of *O*. *volvulus* was widespread in both communities prior to its elimination as a result of intensive mass treatment with Mectizan [2, 12–13]. The local vector, *S*. *ochraceum s*. *l*., is highly anthropophilic and breeding sites surround each study location. Peak productivity of adult flies occurs during the dry season, *i*.*e*., December–May.

### Trap design and deployment

The EWT consists of a 1.0x1.0 m piece of blue plastic tarpaulin supported 0.8 m above ground with a wooden frame, coated with a film of tangle-trap adhesive (Bioquip, Rancho Dominguez, California, USA) and baited with aroma beads saturated with 1-octen-3-ol, 1-octanol, acetophenone, hexanal, and ammonium bicarbonate in roughly equal volumes, as previously described [10]. To complement aerial dispersion of the lures, organically-derived CO_2_ was generated using approximately 17.5 g of Baker’s yeast (*Saccharomyces cerevisiae*) and 250 g of refined sugar dissolved in 2.5 L of purified water. This solution was placed in a 4 L plastic jug and sealed with a stopper in which a piece of plastic tubing was inserted to permit a directed flow of the CO_2_ from the jug to the trap. The jug was placed on the ground next to the trap and the distal end of the tube was affixed to the top of the trap to permit CO_2_ to flow across the trap surface. Baits and yeast solutions were replaced every three days. Two EWTs were deployed daily from 0800 through 1300 h for a total of 10 days at each site. The EWTs were set up in the home and schoolroom, in close proximity of the residents. After 10 days, the traps were removed and fly collections from human subjects were continued from 0800 through 1300 h on the 10 subsequent days. Human landing collectors (HLCs) were placed in the home and in the schoolroom to monitor the biting rate both in the presence and absence of the traps. Each team of collectors was composed of one person serving as an attractant and the other as a collector when flies landed on the attractant. An additional HLC team was also deployed roughly 25m outside the school and household to monitor for fluctuations in the overall fly population during the course of the study. HLCs were carried out from 0800 through 1300 h each day.

**Fig 2:**
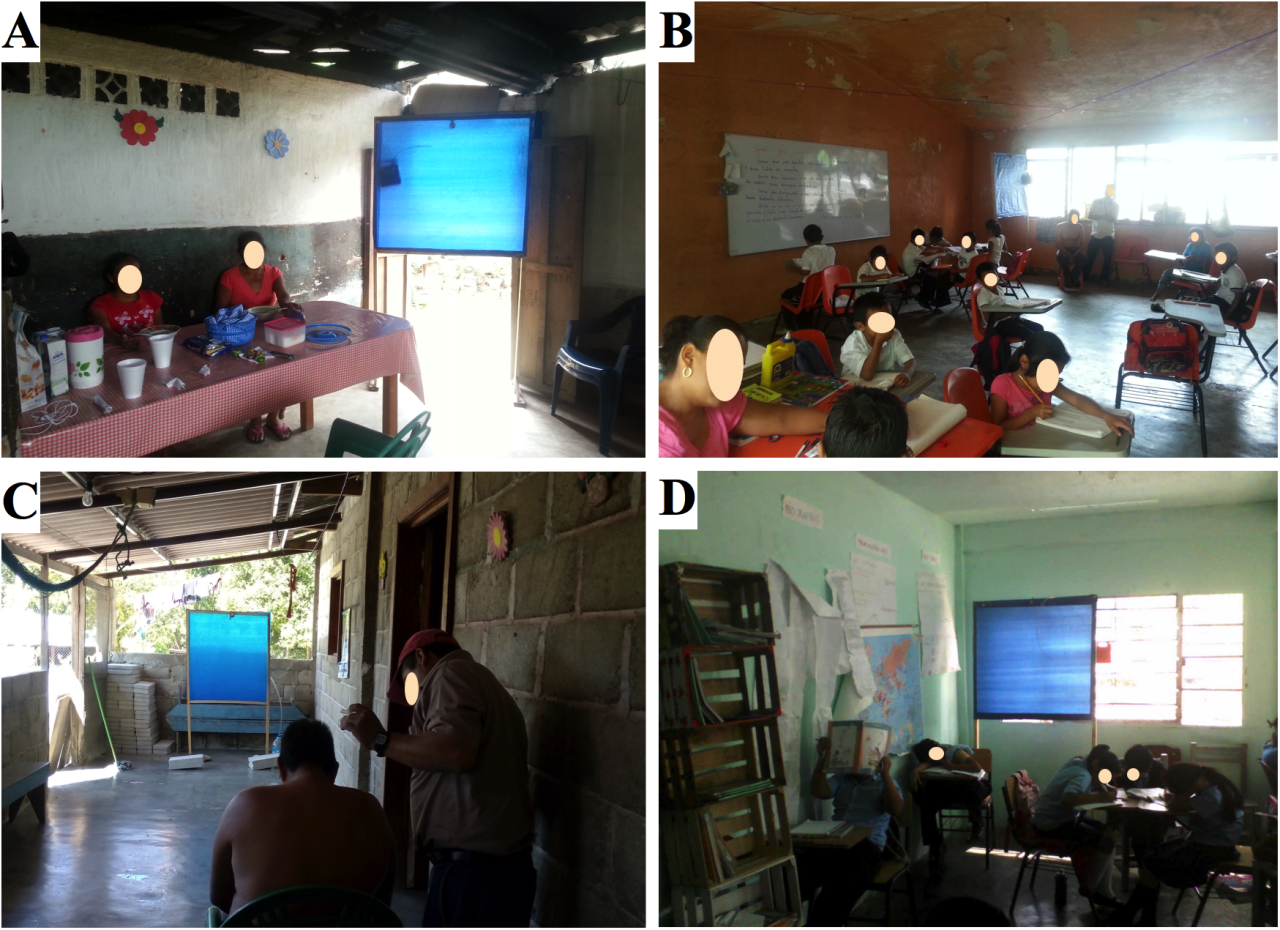
Position of EWT in the households and elementary schools. (A) In Las Golondrinas, the EWTs were deployed at the kitchen-dining area of the household and (B) at the rear of the classroom of the elementary school. (C) In Jose Maria Morelos, the EWTs were deployed at the living room area of the household and (D) at the rear of the classroom of the high school.

In the Las Golondrinas household two EWTs were located near the door to the home. The HLC was located approximately 2m from the traps and there were 5–7 residents present in the home during the course of the study (Fig 2A). In the Las Golondrinas school room the HLCs were carried out approximately 1 to 2 m from the EWTs, which were located at the front of the classroom (Fig 2B). The HLC was positioned in the rear of the classroom and was rotated every day from the left side of the classroom to the right side to minimize disruption of class activities. There were one teacher and 25 children ranging from 7 to 12 years of age in the classroom during the course of the study. In the Jose Maria Morelos household two traps were deployed in the living room area about 5 m from the entrance and exit doors (Fig 2C). Both doors were kept open during trapping experiments. The EWTs were separated about 2 to 3 m from each other. The HLC team inside the household was located approximately 2 m from the EWTs. There were 4 to 5 inhabitants in the household when the experiment was carried out. In the Jose Maria Morelos schoolroom study, two EWTs were set up within the core classroom, which had dimensions of 6 x 5 m (Fig 2D). One trap was placed on the right side and the other on the left side at the rear of the classroom. The HLC team was set up approximately 1 to 2 m from the EWTs. The HLC team was rotated every day from the left side of the classroom to the right and rear sides to minimize disruption of class activities. There were one teacher and 20 children ranging from 12 to 15 years of age in the classroom during the course of the study.

The collection team was the same throughout the surveys to minimize variations resulting from individual differences in attraction and catching ability. Black flies were aspirated from the HLCs as they landed, and were removed from the EWTs at the end of each day using odorless mineral spirits. Collected flies were transferred to 70% ethanol for subsequent morphological identification under stereomicroscopy [9].

### Data analysis

The landing rate measured from the HLCs was used to estimate the human biting rate (number of black flies biting per HLC team/ day). This probably overestimated the human biting rate, as some black flies land but do not successfully take a blood meal. However, the landing rate was chosen since it likely represents the closest approximation of attraction and subsequent biting [2, 12–13]. The generalized linear mixed model (GLIMMIX) in SAS (SAS version 9.4 13w18 Media), was used to fit the number (counts) of black flies caught by the EWTs or HLC to a negative binomial distribution; the least square mean (LSM) of the fly collections in the household, school and outdoors in each community was then calculated as a response variable (RV) in a negative binomial regression and compared between communities [9]. To determine whether daily fly landing collections were correlated among days (*i*.*e*. if the fly collections on the individual days were independent of each other) all time series were examined using the autoregressive integrated moving-average (ARIMA) procedure and chi-square covariance test (Ho: cov = 0) available in proc glimmix for "day". If there was no significant autocorrelation or random effects (cov = 0.03, p > 0.05), then we did not include "day" as an explanatory variable and considered no interaction effect in the final negative binomial regression [14].

To control for variations in the local fly populations the fly collections were corrected by calculating the ratio of the number of flies collected by the HLCs located indoors divided by the number collected by the collectors located outdoors. The statistical significance of differences noted in the corrected collections in the presence and absence of the EWTs was determined using the Student t- test for independent samples. Prior to conducting these tests, the normality of the data was confirmed using the Shapiro-Wilk test in SAS.

## Results

The preponderance of the black flies collected (≥95%) was *S*. *ochraceum s*. *l*., formerly the local vector species. The fly counts from both the HLCs (total = 51,276) and the traps (total = 5,849) were found to conform to a negative binomial distribution, and were thus examined using a generalized linear mixed model. For both studies, the model **No. black flies caught = locality + site + treat** (where treat = flies caught by HLC) was found to fit the data well with no evidence of over-dispersion [15]. There was no evidence of covariance by "day" (covariance value = 0.03); therefore, covariance was not significant by "day". An autocorrelation analysis was then run in SAS on each of the 16 series of data (four per community per site). No significant auto-correlation for the variable No. of flies caught per day was noted in each out of 16 series of data (consisting of 10 days per series; p > 0.05).

**Fig 3:**
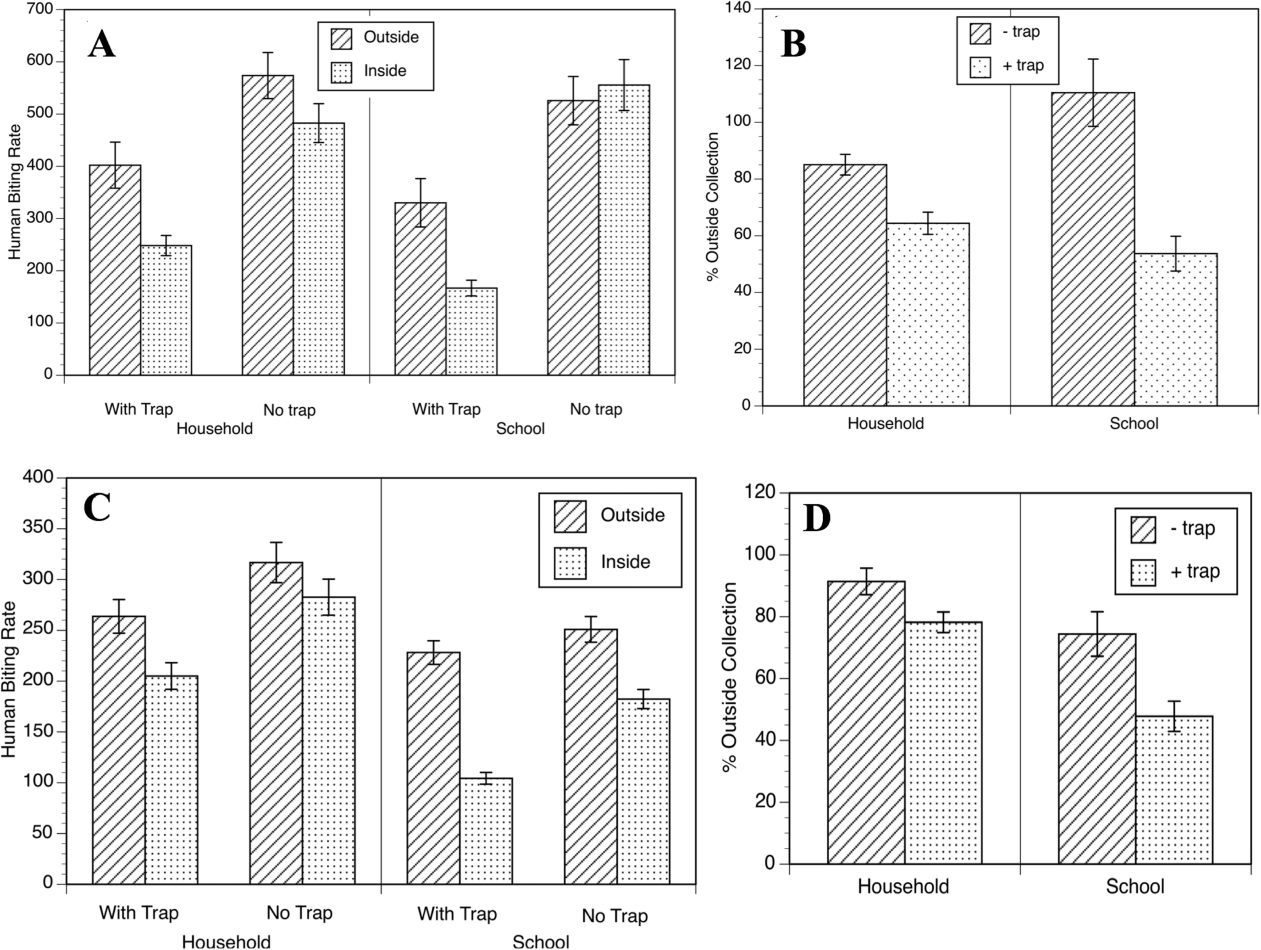
The biting rate of *S*. *ochraceum* s.l. in the household and elementary school of Las Golondrinas and Jose Maria Morelos. (A) Human biting rates indoors in the presence (bars with diagonal lines) and absence (bars with points) of the EWTs. Human biting rate was calculated as the LSM of the number of flies per HLC team per day ± SE. (B) Outdoor collections in the presence (bars with diagonal lines) and absence (bars with points) of the EWTs to normalized normalized to the outdoor biting rate. (C) Human biting rates indoors in the presence (bars with diagonal lines) and absence (bars with points) of the EWTs. Human biting rate was calculated as the LSM of the number of flies per HLC team per day ± SE. Fig (D) Outdoor collections in the presence (bars with diagonal lines) and absence (bars with points) of the EWTs to normalized normalized to the outdoor biting rate.

The LSM of *S*. *ochraceum* s.l. collected/human/day (or biting rate per day) collected by the HLC team in the household in Las Golondrinas was significantly lower in the household when the traps were present than when the traps were absent (Fig 3A). When corrected for variations in the fly population over the course of the study (as measured by the collections obtained by the HLC located outdoors in each community), HLC collections in the household were 85.1 ± 3.6% of those outside the home in the absence of the traps; this was reduced to 64.4 ± 3.9% of the outdoor collections in the presence of the traps (t-value = 3.88; p = 0.0011). This represented a reduction of 24% in the corrected biting rate (Fig 3B).

A similar situation was seen in the Las Golondrinas elementary school. When corrected to the number of flies collected outdoors, the indoor collections in the school were 110.5 ± 11.8% of the outdoor collections in the absence of the traps; this was reduced to 53.8 ± 6.1% of the outdoor collections in their presence (t-value = 4.25; p = 0.0005). This represented a 51% reduction in the corrected biting rate (Fig 3A and 3B).

Field data from Jose Maria Morelos were similar to those from Las Golondrinas. HLC collections in the household (Fig 3C and 3D) were 91.4 ± 4.3% of the outdoor collection in the absence of the traps; this was reduced to 78.3 ± 3.3% of the outdoor collection in their presence (t-value = 2.43; p = 0.0258). This represented a reduction of 14% in the biting rate in the indoor collection in the presence of the traps (Fig 3C and 3D). In the school a more dramatic reduction in the biting rate was observed, as was seen in Las Golondrinas (Fig 3C and 3D). The indoor collections averaged 74.4 ± 7.18% of the outdoor collections in the absence of the traps; this was reduced to 47.8 ± 4.9% in their presence (t-value = 3.06; p = 0.0068). This represented a reduction of 36% in the corrected biting rate (Fig 3D).

## Discussion

Current efforts to eliminate onchocerciasis have relied almost exclusively upon mass drug administration of ivermectin (MDAi), both in Latin America and in Africa with some success [7,14, 16–17]. However, studies have suggested that combining vector control or other measures to reduce human vector contact with MDAi can have a synergistic effect, resulting in more rapid elimination of onchocerciasis. For example, combining vector control with MDAi has been quite successful in rapidly interrupting the transmission of *O*. *volvulus* in several foci in Uganda [18–20]. Furthermore, while the accomplishments in Uganda show that combining MDA and vector control is a very powerful way to rapidly interrupt transmission, in some areas of Africa where transmission is very intense such a combination will be required to achieve elimination. This is due to the fact that transmission intensity is driven by the amount of human exposure to the vector, as measured by the annual biting rate (ABR), or the average number of bites an individual receives over one year. In areas where vector density is very high, annual MDAi alone may not be sufficient to interrupt transmission [21]. Using data from earlier field studies [22–23], stochastic modeling studies have determined that in certain savanna areas of Africa, the threshold human biting rate necessary to maintain an *O*. *volvulus* population is roughly 700–730 bites/year [24]. Due to the impact of annual MDAi treatments on the microfilarial population (community microfilarial load), the required number of bites per person per year necessary to produce sufficient numbers of *O*. *volvulus* L_3_s to maintain the parasite population is shifted upward. Nonetheless, modeling work has suggested that MDA alone may not be sufficient to interrupt transmission in areas where the biting rate is very high [25] and additional measures to reduce human vector contact will likely be required to reduce the biting rate in order for annual MDAi to interrupt transmission. In such areas, combining measures to reduce human vector contact with MDAi to reduce human biting rates to below threshold biting levels as drug pressure increases could lead to elimination of transmission.

The measures that have historically been used to assist in onchocerciasis control have relied upon insecticide treatment of aquatic breeding sites to kill larvae. This approach is often expensive and logistically difficult to accomplish. The results presented above suggest that the EWT may represent a viable control tool by reducing the human biting rate in heavily frequented areas with the greatest potential for contact between human and black flies. The EWT was originally developed as an alternative to HLCs for entomological surveillance to confirm the interruption of transmission of *O*. *volvulus* [8–10,13]. The data presented above suggest that EWTs, when placed in dwellings, can significantly reduce the indoor biting rate of *S*. *ochraceum* s.l., the major vector in the former onchocerciasis foci in México. Thus, it is possible, given the effectiveness and adaptability of the EWTs, that they may serve as an important tool to reduce the human biting rate. Given that the effect of the EWTs will be localized, it is unlikely that they will capture sufficient numbers of flies to reduce the vector population overall. Thus, the effect of the EWTs is likely to be more similar to personal protective devices (*i*.*e*. screens, insect repellants or indoor insecticide treatments) than to classical vector control measures, such as larvacide treatment of breeding sites.

While these results are encouraging, more work will be needed before the EWT will become an effective measure of reducing human vector contact. First and most importantly, the EWT must be shown to be effective in reducing the human biting rate when deployed against *S*. *damnosum* s.l., the major African vector of *O*. *volvulus*. While the EWT has been shown to be as effective as a HLC team for the collection of *S*. *damnosum* s.l. for entomological surveillance purposes [10], it is not known if when deployed it will significantly reduce the local biting rate below threshold rates necessary to maintain *O*. *volvulus* transmission when combined with MDAi [20]. Furthermore, the behavior of *S*. *damnosum* s.l. differs from that of *S*. *ochraceum* s.l., with the former tending to bite most frequently in outdoor locations, including near its riverine breeding sites and in fields [26–27]. The effectiveness of the EWT in reducing *S*. *damnosum* s.l. biting in such outdoor locations (when people will not be located as near to the trap as they are in the indoor locations tested here) will have to be evaluated. Finally, for the EWT to be economically viable as a vector control measure, it will be necessary for the traps to be operated and maintained by members of the afflicted communities. Previous studies that have shown that communities are capable of operating the EWT as an entomological surveillance tool [9] suggest that such community-based operation will be feasible.

## Supporting information

S1 Data Raw data of daily no. of flies caught by a team of human landing collectors in the presence and absence of the traps. (XLSX)
